# The Haploinsufficient Hematopoietic Microenvironment Is Critical to the Pathological Fracture Repair in Murine Models of Neurofibromatosis Type 1

**DOI:** 10.1371/journal.pone.0024917

**Published:** 2011-09-29

**Authors:** Xiaohua Wu, Shi Chen, Yongzheng He, Steven D. Rhodes, Khalid S. Mohammad, Xiaohong Li, Xianlin Yang, Li Jiang, Grzegorz Nalepa, Paige Snider, Alexander G. Robling, D. Wade Clapp, Simon J. Conway, Theresa A. Guise, Feng-Chun Yang

**Affiliations:** 1 Department of Pediatrics, Indiana University School of Medicine, Indianapolis, Indiana, United States of America; 2 Herman B Wells Center for Pediatric Research, Indiana University School of Medicine, Indianapolis, Indiana, United States of America; 3 Anatomy and Cell Biology, Indiana University School of Medicine, Indianapolis, Indiana, United States of America; 4 Pediatric Hematology-Oncology, Indiana University School of Medicine, Indianapolis, Indiana, United States of America; 5 Endocrinology and Metabolism, Department of Internal Medicine, Indiana University School of Medicine, Indianapolis, Indiana, United States of America; University of Medicine and Dentistry of New Jersey, United States of America

## Abstract

Germline mutations in the *NF1* tumor suppressor gene cause neurofibromatosis type 1 (NF1), a complex genetic disorder with a high predisposition of numerous skeletal dysplasias including short stature, osteoporosis, kyphoscoliosis, and fracture non-union (pseudoarthrosis). We have developed murine models that phenocopy many of the skeletal dysplasias observed in NF1 patients, including reduced bone mass and fracture non-union. We also show that the development of these skeletal manifestations requires an *Nf1* haploinsufficient background in addition to nullizygous loss of *Nf1* in mesenchymal stem/progenitor cells (MSCs) and/or their progenies. This is replicated in two animal models of NF1, *PeriCre^+^;Nf1^flox/−^* and *Col2.3Cre^+^;Nf1^flox/−^*mice. Adoptive transfer experiments demonstrate a critical role of the *Nf1+/−* marrow microenvironment in the impaired fracture healing in both models and adoptive transfer of WT bone marrow cells improves fracture healing in these mice. To our knowledge, this is the first demonstration of a non-cell autonomous mechanism in non-malignant NF1 manifestations. Collectively, these data provide evidence of a combinatory effect between nullizygous loss of *Nf1* in osteoblast progenitors and haploinsufficiency in hematopoietic cells in the development of non-malignant NF1 manifestations.

## Introduction

Bone integrity is maintained by the balance between osteoclasts originated from hematopoietic stem cells and mesenchymal-derived osteoblasts. Osteoclasts are multinucleated cells specialized in bone resorption, whereas osteoblasts are bone forming cells derived from the mesenchymal stem cells. The development of the osteoblast and osteoclast lineages is tightly coupled [1]. Imbalances in coupling of the two lineages leads to defective skeletal development [2] as well as abnormal hematopoiesis [3], [4].

Mutations in the *NF1* tumor suppressor gene cause neurofibromatosis type 1 (NF1), a common chronic debilitating disorder that affects 1 in 3,000 individuals [5], [6], [7], [8], [9]. Neurofibromin, the protein encoded by *NF1*, functions at least in part as a GTPase activating protein (GAP) for Ras. Loss of heterozygosity of the tumorigenic cell is required for genesis of tumors in individuals with NF1 [10], [11]. However, recent studies have also identified a key role of the microenvironment in tumor progression in both plexiform neurofibromas [12] and optic gliomas [13], [14] in NF1. Despite advances in understanding the cell-cell interactions in cancers affecting NF1 patients, little information of the molecular and cellular signaling of the microenvironment of the prevalent nonmalignant manifestations of NF1 exists, particularly those impacting the skeletal system that are prevalent in ∼50% of NF1 patients [6], [7], [8], [9], [15], [16], [17], [18], [19], [20]. Clinical studies have found that individuals with NF1 are at significant risk for generalized loss of bone mass (osteopenia and osteoporosis) [21], [22], [23], [24], [25], [26] as well as focal skeletal abnormalities [25], [27], [28], [29]. One focal skeletal abnormality, non-union of spontaneous fractures, also called pseudoarthrosis, is particularly debilitating and frequently affects the distal tibia. Despite a range of orthopedic procedures attempting to facilitate fracture healing, the typical clinical outcome is non-union of the fracture and ultimately amputation of the affected limbs.

Skeletal development and remodeling depends on the balance of interactions between osteoblasts-mesenchymal stem cell derived cells, and osteoclasts-specialized macrophages derived from hematopoietic stem cells that govern skeletal remodeling and bone resorption [30]. We have previously shown that NF1 patients and *Nf1* haploinsufficient mice have elevated osteoclast activity [31]. However, the increase in lytic activity of osteoclasts alone is not sufficient to explain some of the osseous manifestations observed in the NF1 patients. Kolanczyk and colleagues observed bowing of the tibia and diminished growth in *Nf1^flox/flox^* mice that express Prx1Cre, which is expressed in undifferentiated mesenchymal cells in the limb bud. While those studies were informative concerning limb manifestations of the disease, the axial skeletal (e.g. kyphosis) are not modeled well due to a lack of expression of PrxCre in the vertebrae [32]. Elefteriou and colleagues utilized a genetic approach to delete *Nf1* in the osteoblast lineage by intercrossing *Nf1^flox/flox^* mice with *α1(I)* Collagen *Cre* transgenic mice and found that *Nf1* inactivation in osteoblasts affects osteoclast differentiation in addition to bone formation [33], [34].

In the present study, we utilized two novel murine models and established that the hematopoietic microenvironment plays a critical role in fracture healing in the NF1 murine models. Our study provides strong evidence of a non-cell autonomous mechanism for the skeletal manifestations of NF1 and demonstrates that the hematopoietic microenvironment is instrumental for bone loss and impaired fracture healing process.

## Results

### Detection of periostin expression in MSCs, osteoblasts, and generation of *PeriCre^+^;Nf1^flox/−^* mice

Endogenous full-length Periostin is expressed in primitive MSCs and epithelial cells [35], [36]. To confirm 3.9 kb Periostin fragment promoter mediated Cre expression in adult MSCs and skeletal tissues, *PeriCre^+^* mice were intercrossed with transgenic reporter mice that have LacZ sequence knocked into the Rosa26 locus. The skeletal tissues of the intercrossed progeny were then subjected to β-gal staining. LacZ expression was abundant in the peripheral nervous system as expected [37] but was also detected in osteoblasts within the cartilaginous ribs as they begin the process of ossification, the long bone, and vertebrae cartilage of adult *PeriCre^+^*;Rosa26*^flox/flox^* mice (data not shown). However, LacZ staining was not observed in *PeriCre^−^*;Rosa26*^flox/flox^* littermates (Figure S1A, c, d). The expression of LacZ in bone tissue was confirmed following LacZ staining and hematoxylin/eosin (H&E) counterstaining (Figure S1A). Interestingly, LacZ expression within MSCs and skeletal tissues was detected only in mice 4 weeks of age or older, as *PeriCre* activity is initially restricted to the *in utero* heart valves, fetal and neonatal cardiac fibroblasts [38], and peripheral nervous system (Snider & Conway, unpublished data). Significantly, like the endogenous Periostin protein, *Peri*-Cre reporter expression is robustly localized to both the postnatal and adult outer periosteum, which gives rise to the bone collar covering all skeletal elements, including cancellous bone, and within the inner endosteal osteoblasts that secrete the vascularized bone-specific matrix [39]. LacZ expression was also examined in adult primary calvarial osteoblasts and MSCs *in vitro* following culture in specialized medium that selectively promotes the growth of these lineages. LacZ was strongly expressed in cultured adult calvarial osteoblasts and ∼15% of the cultured MSCs were LacZ positive (Figure S1B, a, b).

*PeriCre^+^* mice were then intercrossed with mice bearing either systemic or conditional genetic targeting of the *Nf1* allele (*Nf1+/−* and *Nf1^flox/flox^*, respectively) to generate four experimental genotypes: *PeriCre^−^;Nf1^flox/flox^* (control, referred to as WT mice), *PeriCre^−^;Nf1^flox/−^* (referred to as *Nf1+/−*), *PeriCre^+^;Nf1^flox/flox^* mice (*Nf1* null in MSCs and osteoblasts, otherwise systemically WT), and *PeriCre^+^;Nf1^flox/−^* mice (*Nf1* null in MSCs and osteoblasts, otherwise systemically *Nf1* heterozygous). The offspring were genotyped by PCR (Figure S2). The postnatal deletion of neurofibromin protein was confirmed by western blot. A clear gene dose-dependent reduction of neurofibromin was detected in *Nf1+/−* MSCs and osteoblasts as compared to their WT counterparts and no neurofibromin was detected in adult *Nf1−/−* MSCs and osteoblasts (Figure S1C).

### *PeriCre^+^;Nf1^flox/−^* mice have reduced bone mineral density (BMD) and bone volume

To evaluate whether the *PeriCre^+^;Nf1^flox/−^* mouse model recapitulates the bone manifestations of NF1 patients, we measured skeletal mass and density in the four experimental genotypes. *PeriCre^+^;Nf1^flox/−^* mice and *PeriCre^+^;Nf1^flox/flox^* mice were born at the expected Mendelian ratio. Interestingly, syngeneic adult *PeriCre^+^;Nf1^flox/−^* mice were smaller than the other three groups of mice (Figure S3A). Furthermore, *PeriCre^+^;Nf1^flox/−^* mice had reduced body weight and length as compared to WT, *Nf1+/−*, or *PeriCre^+^;Nf1^flox/flox^* mice (Figure S3B, C).

**Figure 1:**
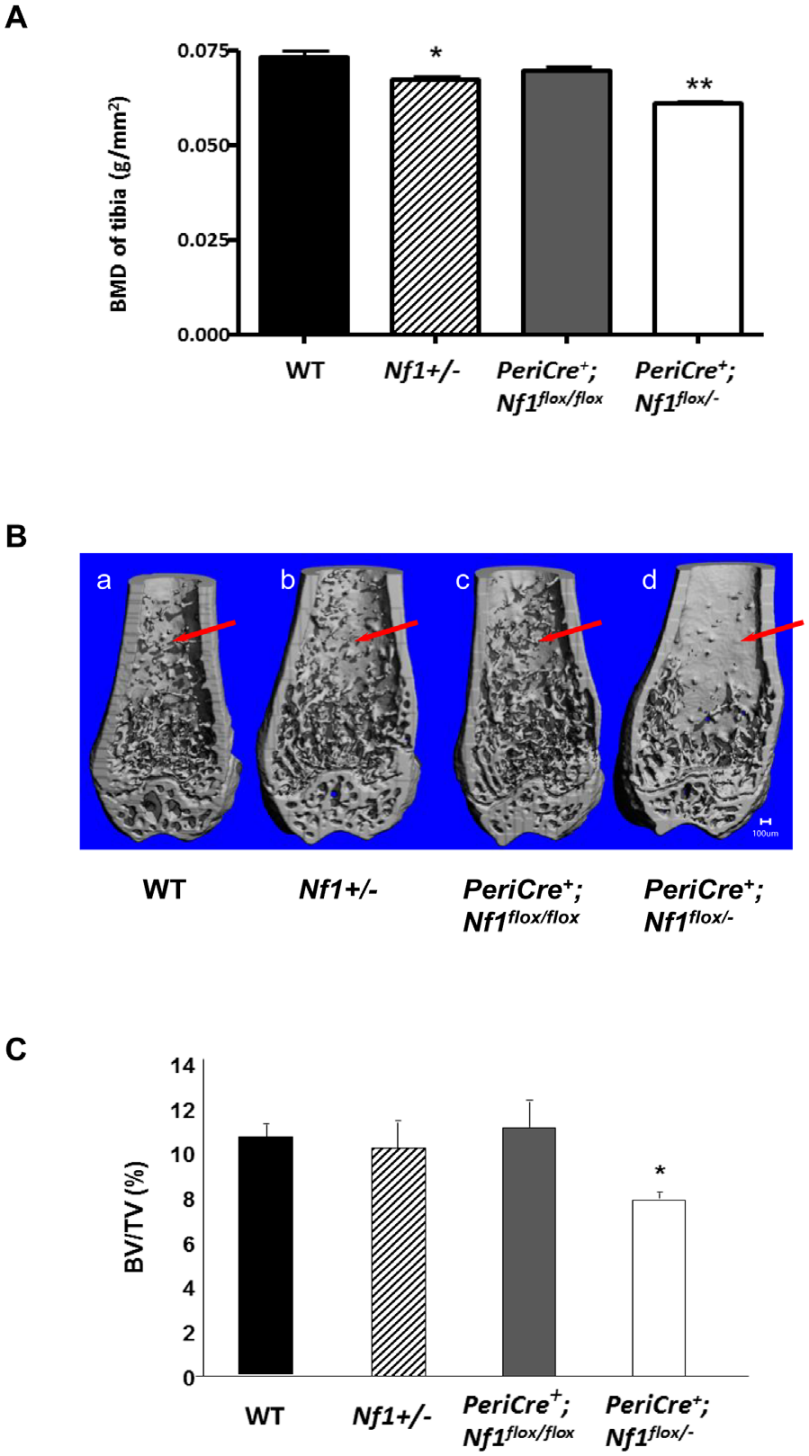
Loss of *Nf1* results in reduced bone mineral density (BMD), volume (BV), and trabecular architecture *in vivo*. (**A**) pDEXA scans on 4–6-month-old mice revealed a 22% reduction of whole body BMD in *PeriCre^+^;Nf1^flox/−^* mice as compared to the other 3 genotypes of mice (n≥9/group, * and ** P<0.05). (**B**) μCT scans through the distal femur of 16-wk old mice revealed a significant reduction of trabecular bone volume in the *PeriCre^+^;Nf1^flox/−^* mice. The anterior portion of each femur had been removed for the metaphyseal architecture analysis. Red arrows indicate the deficiency in the trabecular network in the *PeriCre^+^;Nf1^flox/−^* mice vs. controls. (**C**) Quantitation of the trabecular bone volume reveals an ∼22% reduction of bone volume per unit total volume (BV/TV) in the distal femoral metaphysis of *PeriCre^+^;Nf1^flox/−^* mice as compared to the other genotypes (n = 4–6/group, * and ** P<0.05).

To investigate these skeletal phenotypes in more detail, a series of radiographic analyses were conducted to measure BMD and bone architecture. *PeriCre^+^;Nf1^flox/−^* mice were shown to have reduced whole body BMD as compared to *PeriCre^+^;Nf1^flox/flox^*, WT, or *Nf1+/−* mice (Figure 1A) measured by peripheral Dual-Energy X-Ray Absorptiometry (pDEXA). When trabecular bone of 16-week old mice were evaluated using micro-computed tomography (μCT), the bone volume (BV/TV) was similar in WT, *Nf1+/−*, and *PeriCre^+^Nf1^flox/flox^* mice, while *PeriCre^+^Nf1^flox/−^* mice exhibited an ∼23% reduction as compared to the other three genotypes (Figure 1B–C).

### *PeriCre^+^;Nf1^flox/−^* mice have increased osteoclasts, reduced osteoblast formation, and altered bone turnover *in vivo*

**Figure 2:**
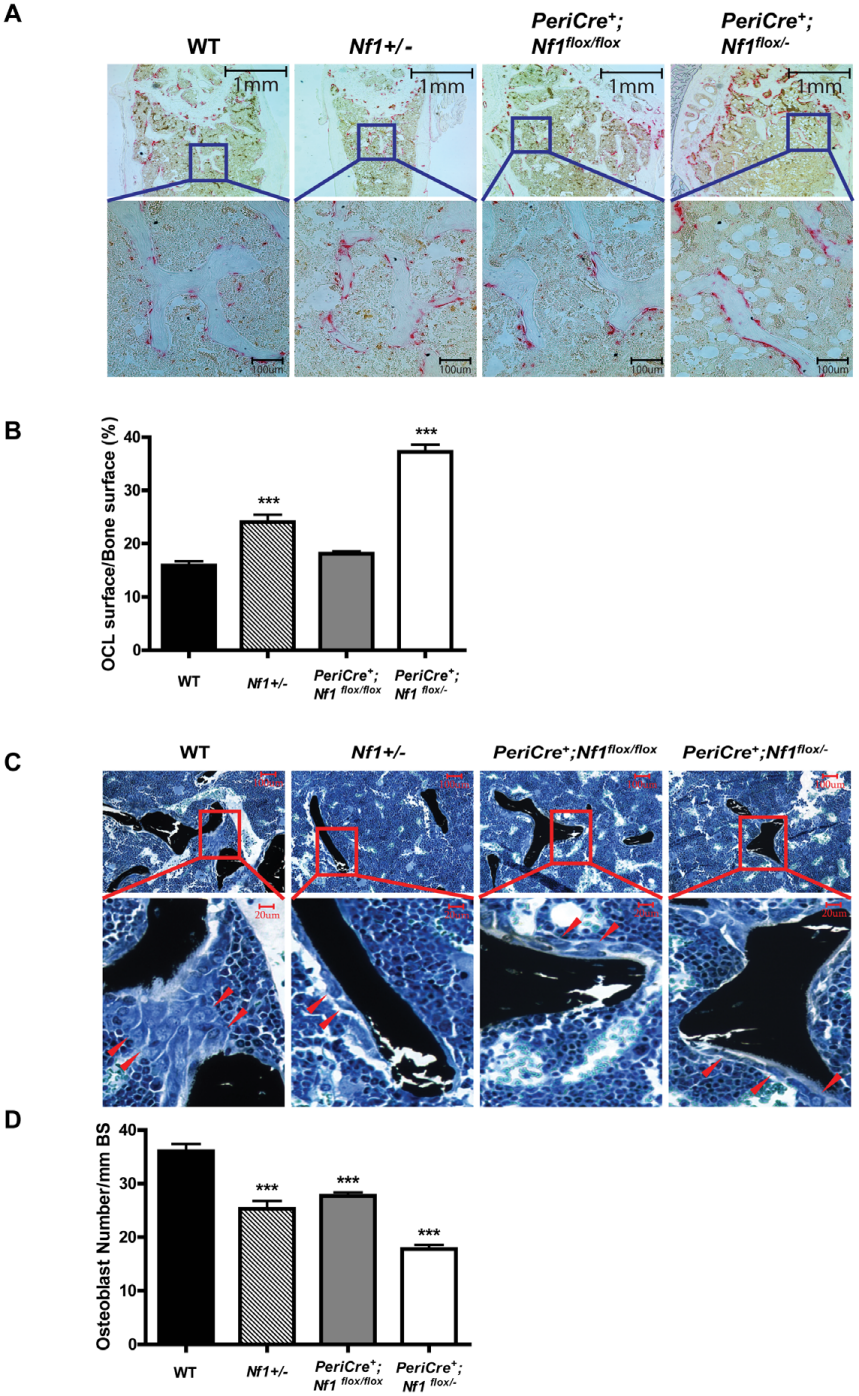
*PeriCre^+^;Nf1^flox/−^* mice have increased osteoclasts and decreased osteoblasts *in vivo*. (**A**) TRACP staining revealed a significant increase of osteoclasts in *PeriCre^+^;Nf1^flox/−^* mice. Representative photomicrographs shows TRACP+ osteoclasts in tibia of each mouse genotype (Red staining are TRACP positive osteoclasts). (**B**) Quantitative data showing the change of surfaces/BS ratio. ***P<0.001 for *Nf1+/−* and *PeriCre^+^; Nf1^flox/−^* VS WT controls. (**C**) Representative photomicrograph showing MacNeal staining of the tibia of each mouse genotype (Red arrow heads are pointing to osteoblasts). (**D**) Quantitative data showing the osteoblast number in trabecular bone/ mm BS. ***P<0.001 for . WT control vs *PeriCre^+^;Nf1^flox/−^* , *Nf1+/−* and *PeriCre^+^;Nf1^flox/flox^* mice.

Biochemical markers of bone turnover are indicative of the overall bone metabolism and provide additional information besides the measurement of BMD [40]. Therefore, we evaluated the osteoclast activity in the trabecular bone of the four genotypes of mice by tartrate-resistant acid phosphatase (TRACP) staining and scoring the TRACP^+^ osteoclasts surface (OcS) in the tibial trabeculae over trabecular bone surface (BS). A significantly greater OcS/BS ratio in the trabecular bone was observed in *Nf1+/−* and *PeriCre^+^;Nf1^flox/−^* mice than WT and *PeriCre^+^;Nf1^flox/flox^* mice as shown qualitatively and quantitatively in Figure 2A and Figure 2B, respectively. Furthermore, *PeriCre^+^;Nf1^flox/−^* mice had significantly higher serum levels of TRACP5b compared to WT mice (1.26 unit±0.06 vs. 1.82unit±0.10, respectively, p<0.005) as measured by ELISA, suggesting an increased osteoclast development *in vivo*. In addition, a significantly lower number of osteoblasts was observed in *Nf1+/−, PeriCre^+^;Nf1^flox/flox^* and *PeriCre^+^;Nf1^flox/−^* mice compared to WT mice, as evaluated by counting osteoblasts in tibial trabecular bone on MacNeal's stained sections (Figure 2C–D). Interestingly, *PeriCre^+^;Nf1^flox/−^* mice had reduced osteoblast numbers in comparison to *PeriCre^+^;Nf1^flox/flox^* mice given that both genotypes of mice had a similar genetic background in mesenchymal-osteoblast lineage. This suggests an additional effect of the haploinsufficient hematopoietic microenvironment on the skeletal deficits in *PeriCre^+^;Nf1^flox/−^* mice *in vivo*.

**Figure 3:**
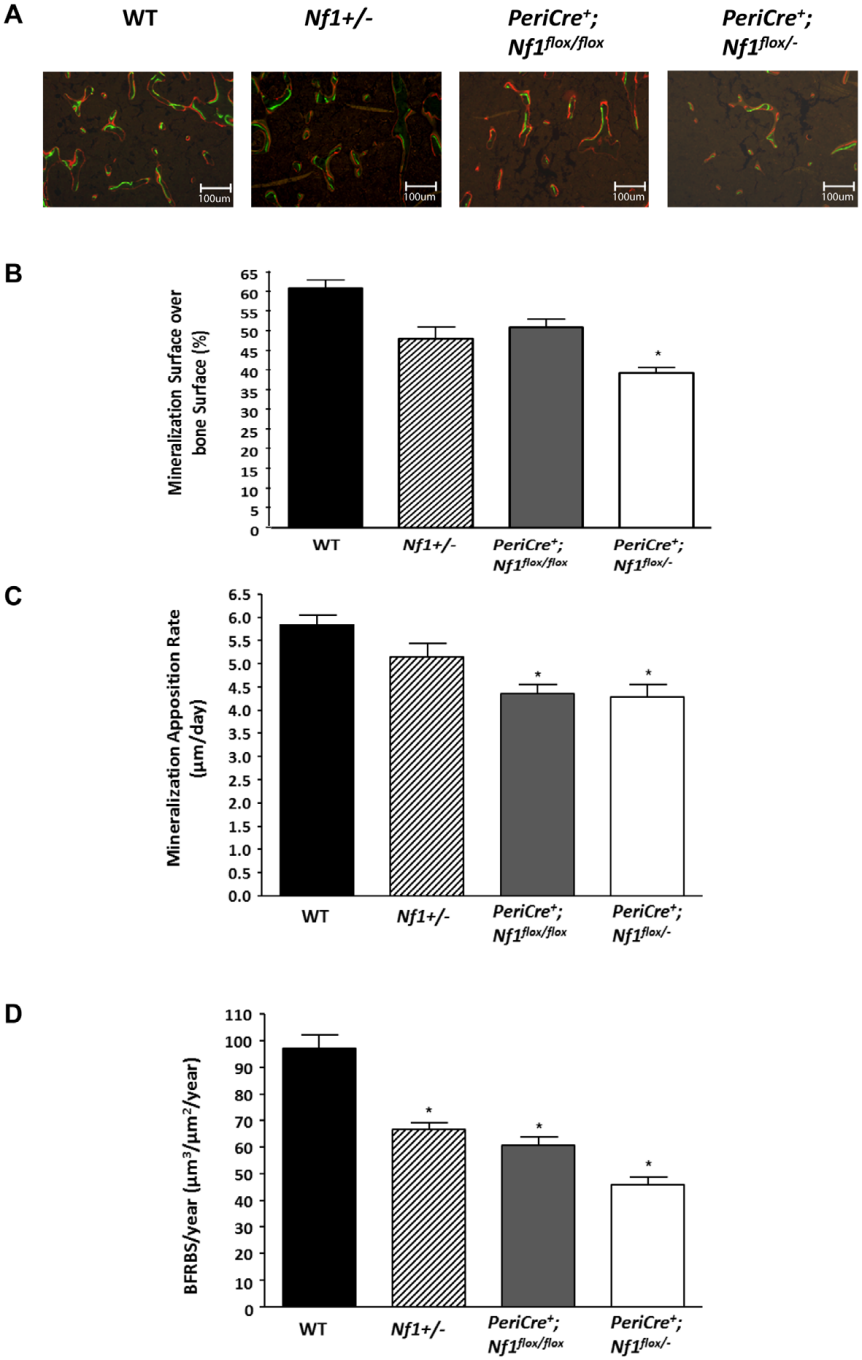
Loss of *Nf1* results in reduced bone turnover *in vivo*. (**A**) Representative photomicrographs showing the uptake of calcein and alizarin fluorochromes into newly mineralizing bone. (**B**) Quantification of the proportion of the total trabecular surface that was actively engaged in bone mineralization (MS/BS). (**C**) The radial rate of bone deposition at active sites (MAR) is shown. (**D**) The total rate of newly forming bone (BFRBS/year) was calculated and shown.

Labeling bones with fluorochrome markers provides a means to study the dynamic changes of bone formation [41]. Thus, we examined bone remodeling in the four genotypes of mice using fluorescent labeling. There was a 30% reduction in mineralizing surface (MS/BS), a 20% reduction in mineral apposition rate (MAR), and a 50% reduction in bone formation rate (BFR/BS) in *PeriCre^+^;Nf1^flox/−^* mice in comparison to control mice (Figure 3A–D). Together, these data suggest that *PeriCre^+^;Nf1^flox/−^* mice had a significant reduction in bone turnover in comparison to WT, *Nf1+/−*, or *PeriCre^+^;Nf1^flox/flox^* mice. The observation that skeletal phenotypes manifest in adult *PeriCre^+^;Nf1^flox/−^* mice, but not *PeriCre^+^;Nf1^flox/flox^* or *Nf1+/−* mice, suggests that the development of skeletal defects requires nullizygous loss of *Nf1* in MSCs/osteoblasts and haploinsufficient loss of *Nf1* in other cell lineages.

### *PeriCre^+^;Nf1^flox/−^* mice have delayed fracture healing

**Figure 4:**
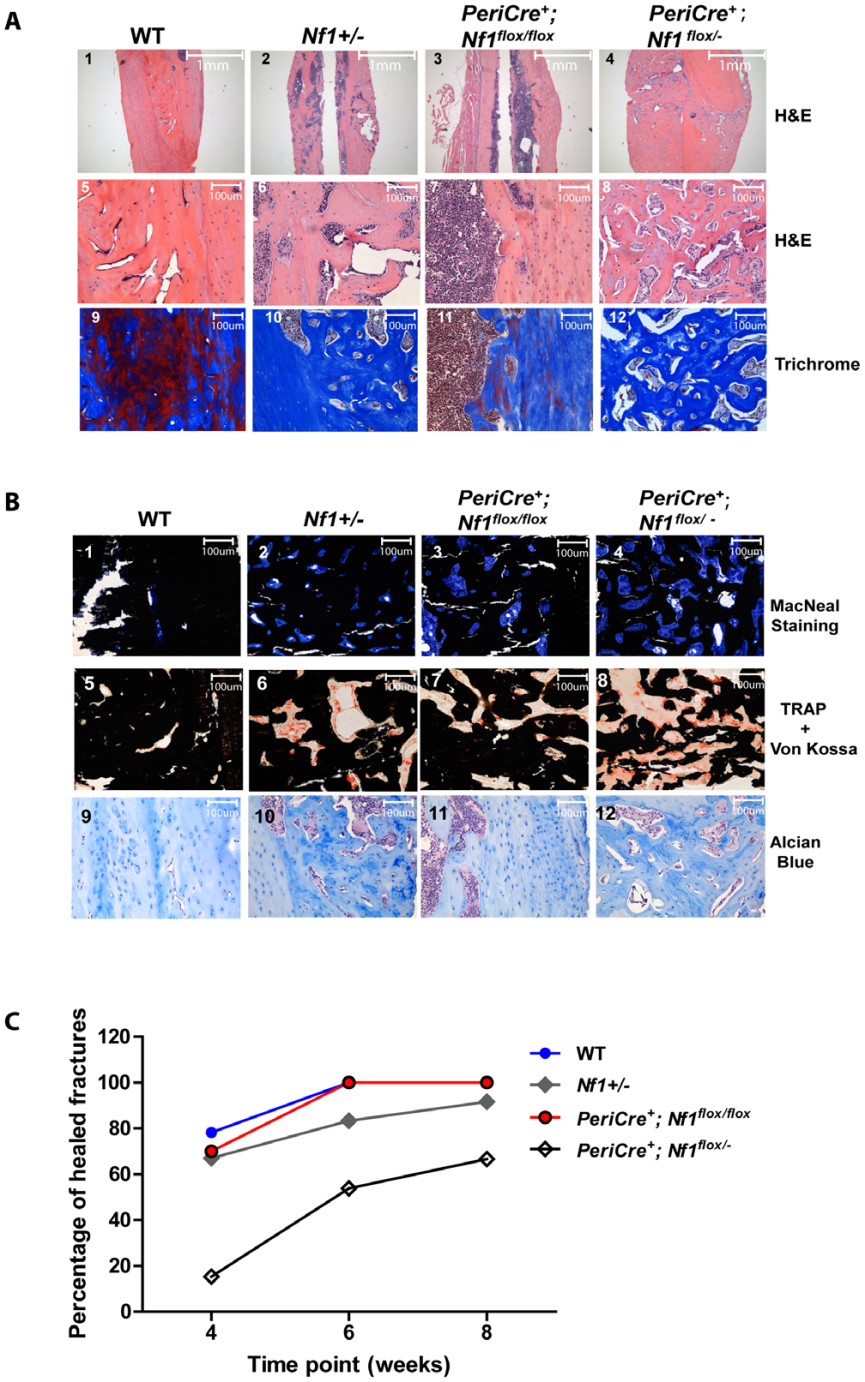
*PeriCre^+^;Nf1^flox/−^* mice have a delayed fracture healing process. (**A**) H&E staining (upper and middle panels 1–4, 5–8) and Masson Trichrome staining (lower panels10–12). (**B**) McNeal/von Kossa staining (upper panels1–4), TRACP/Von Kossa (middle panels 5–8), and Alcian blue staining (lower panels 9–12) of the tibia 4 weeks after the fracture. (**C**) Percent of healed fractures at a series of time points.

Approximately 3% of young children with NF1 acquire pseudoarthrosis [42]. To assess whether the fracture healing process is altered in *PeriCre^+^;Nf1^flox/−^* mice, we induced three point bending fractures in the control and experimental mice. WT mice healed 28 days after the fracture as evaluated by X-ray (data not shown) and histological analysis following H&E, Masson Trichrome, McNeal staining and TRACP/Von Kossa staining (Figure 4 A, B). In contrast, only 15% of *PeriCre^+^;Nf1^flox/−^* mice had healed fractures after 28 days. While WT mice had a normal architecture in the fractured tibia, *Nf1+/−* and *PeriCre^+^;Nf1^flox/−^* tibias showed deficient trabecular and cortical bone formation and increased osteoclasts in the callus site and impairment was most severe in *Peri^Cre+^;Nf1^flox/−^* mice (Figure 4A, B). These data are summarized in Figure 4C. To evaluate whether the fractures in *PeriCre^+^;Nf1^flox/−^* mice are delayed in healing or non-union, the fracture healing time was extended to 8 weeks after the fractures. As shown in Figure 4C, 33% of the *PeriCre^+^;Nf1^flox/−^* mice withstood the non-unioned fractures at the end of 8-weeks.

### *Col2.3Cre^+^;Nf1^flox/−^* mice have reduced bone mineral density (BMD) and reduced bone volume

**Figure 5:**
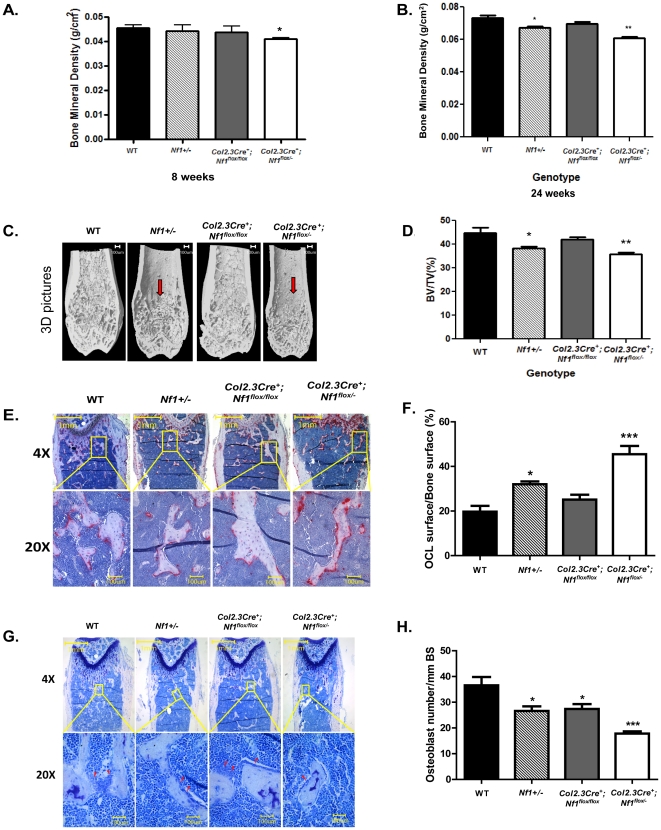
Loss of *Nf1* results in reduced bone mineral density (BMD), volume (BV), and trabecular architecture in *Col2.3Cre^+^;Nf1^flox/−^* mice *in vivo*. BMD was measured with pDEXA scans at 8 weeks (**A**) (*p<0.05) and 24 weeks (**B**) (*p<0.05, **p<0.01). WT (n = 12), *Nf1+/−* (n = 10), *Col2.3Cre^+^;Nf1^flox/flox^* (n = 12), and *Col2.3Cre^+^;Nf1^flox/−^* (n = 11). (**C**) Micro-CT (μCT) scans were performed on the distal femurs of 4 month old WT (n = 5), *Nf1+/−* (n = 5), *Col2.3Cre^+^;Nf1^flox/flox^* (n = 5) and *Col2.3Cre^+^;Nf1^flox/−^* (n = 5) mice. Representative reconstructed 3D pictures are shown. The anterior portion of each femur has been removed for the metaphyseal architecture analysis. Deficiencies in the trabecular network are indicated by the red arrows. (**D**) Quantitation of 3D reconstruction and BV/TV ratio analysis. Trabecular bone formation is decreased in both *Nf1+/−* (*p<0.05) and *Col2.3Cre^+^;Nf1^flox/−^* (**p<0.01) mice as compared to WT and *Col2.3Cre^+^;Nf1^flox/flox^* mice. Four month old WT (n = 5), *Nf1+/−* (n = 5), *Col2.3Cre^+^;Nf1^flox/flox^* (n = 5), and *Col2.3Cre^+^; Nf1^flox/−^* (n = 5) mouse femur samples were processed for analysis. TRACP staining (**E**, **F**) and MacNeals' staining (**G**, **H**) were performed. (**E**) Representative photographs of the trabecular bone following TRACP staining (upper panel 4× and lower panel 20×) are shown. The red stained area indicates osteoclasts. (**F**) Quantitative analysis showing the change of osteoclast surface per bone surface. ***p<0.0001 as analyzed by ANOVA and Bonferroni's Multiple Comparison Test. (**G**) Representative photomicrographs (upper panel 4× and lower panel 20×) are shown. Light pink staining represents bony tissue (trabecular spicules). Blue staining represents the marrow cavity and includes bone marrow cells. Red arrow head are pointing to osteoblasts. (**H**) Quantitative data of osteoblast number over bone surface. One-way ANOVA analysis and Bonferroni's Multiple Comparison Test were used to evaluate statistical significance of the results. ***p<0.0001.

Since the expression of periostin Cre is in both MSCs and osteoblasts, we sought to establish whether *Nf1* deficiency, specifically in the osteoblast lineage, would result in similar phenotypic skeletal manifestations. Conditional bi-allelic deletion of *Nf1* in osteoblasts was achieved by genetic intercross of *Nf1^flox/flox^* mice with *Col2.3Cre+* mice, placing the *Cre* gene under the control of the 2.3-kb proximal fragment of the α1(I)-collagen promoter, which is specifically expressed in osteoblasts [33], [43]. All genotypes of mice, including WT, *Nf1+/−*, *Col2.3Cre^+^;Nf1^flox/flox^* , and *Col2.3Cre^+^;Nf1^flox/−^* mice, were born at the expected Mendelian ratio. Similar to *PeriCre^+^;Nf1^flox/−^* mice, syngeneic adult *Col2.3Cre^+^;Nf1^flox/−^* mice displayed an ∼20% reduction in body weight and a 10% reduction in body length as compared to WT, *Nf1+/−*, or *Col2.3Cre^+^;Nf1^flox/flox^* mice (data not shown). To investigate these skeletal phenotypes in more detail, a series of radiographic analyses were conducted to measure BMD and bone architecture. At 8 weeks of age, *Col2.3Cre^+^;Nf1^flox/−^* mice had an ∼10% reduction in tibial BMD as compared to WT, *Nf1+/−*, or *Col2.3Cre^+^;Nf1^flox/flox^* mice (Figure 5A) as measured by pDEXA. At 24 weeks of age, *Nf1+/−* tibiae showed a 13% reduction in BMD as compared to WT control mice. Yet, an even greater reduction of 22% in BMD was observed in tibiae of *Col2.3Cre^+^;Nf1^flox/−^* mice compared to WT mice (Figure 5B). However, no significant reduction of tibial BMD was found in *Col2.3Cre^+^;Nf1^flox/flox^* mice as compared to WT controls.

To compare the trabecular bone volume and architecture of long bone between the four mouse genotypes, micro-computed tomography (μCT) analysis was performed. *Col2.3Cre^+^;Nf1^flox/−^* mice exhibited an ∼23% reduction in bone volume fraction (BV/TV) as compared to the WT and *Col2.3Cre^+^; Nf1^flox/flox^* mice (Figure 5C–D). *Nf1+/−* mice exhibited an ∼16% reduction in BV/TV as compared to WT and *Col2.3Cre^+^; Nf1^flox/flox^* mice. This reduced bone volume in *Col2.3Cre^+^; Nf1^flox/−^* and *Nf1+/−* mice is consistent with the reduction of BMD in these mice.

### *Col2.3Cre^+^;Nf1^flox/−^* mice have increased osteoclast formation and reduced osteoblast number *in vivo*

We next identified osteoclasts in the distal femoral metaphysis by TRACP staining and scored the ratio of osteoclast surface over trabecular surface of each femur as a measure of osteoclast activity. Interestingly, a significantly greater CS/BS ratio in the trabecular bone was observed in *Nf1+/−* and *Col2.3Cre^+^;Nf1^flox/−^* mice as compared to WT and *Col2.3Cre^+^;Nf1^flox/flox^* mice (Figure 5E–F). The significant difference in CS/BS ratio was even more dramatic in *Col2.3Cre^+^;Nf1^flox/−^* mice. In addition, a significantly lower osteoblast number per millimeter trabecular bone surface was observed in *Nf1+/−, Col2.3Cre^+^;Nf1^flox/flox^* and *Col2.3Cre^+^;Nf1^flox/−^* mice as compared to WT mice (Figure 5G–H). Interestingly, *Col2.3Cre^+^;Nf1^flox/−^* mice had lower osteoblast number than *Col2.3Cre^+^;Nf1^flox/flox^* mice, even though both genotypes had identical *Nf1* alleles in the osteoblast lineage, suggesting the potential of an additional effect of the haploinsufficient hematopoietic microenvironment on bone formation *in vivo*.

### *Nf1+/−* bone marrow transplantation impairs fracture healing in *Nf1^flox/flox^;PeriCre+* mice

To formally test the hypothesis that the hematopoietic microenvironment has a key role in fracture healing in *PeriCreNf1^flox/flox^* mice, bone marrow mononuclear cells (BMMNCs) from either WT or *Nf1+/−* donor mice, that also express an EGFP transgene, were transplanted into *PeriCre^+^;Nf1^flox/−^*, *PeriCre;Nf1 ^flox/flox^* recipient mice that had been previously treated with ionizing radiation [31]. The hematopoietic reconstitution was verified with flow cytometric analysis demonstrating that the reconstituted mice had >95% EGFP positive donor bone marrow cells and clonogenic myeloid progenitors (Figure S4). To clarify whether the donor mesenchymal stem cells were reconstituted in the recipient bone marrow, we cultured CFU-F using the recipient bone marrow cells. Two weeks later, we scored the CFU-F under the fluorescent microscope. All the CFU-Fs were negative in GFP, suggesting the mesenchymal stem cells were originated from recipients, but not the donor cells. This data indicated that the donor hematopoietic cells are key for the skeletal changes in the recipient mice.

**Figure 6:**
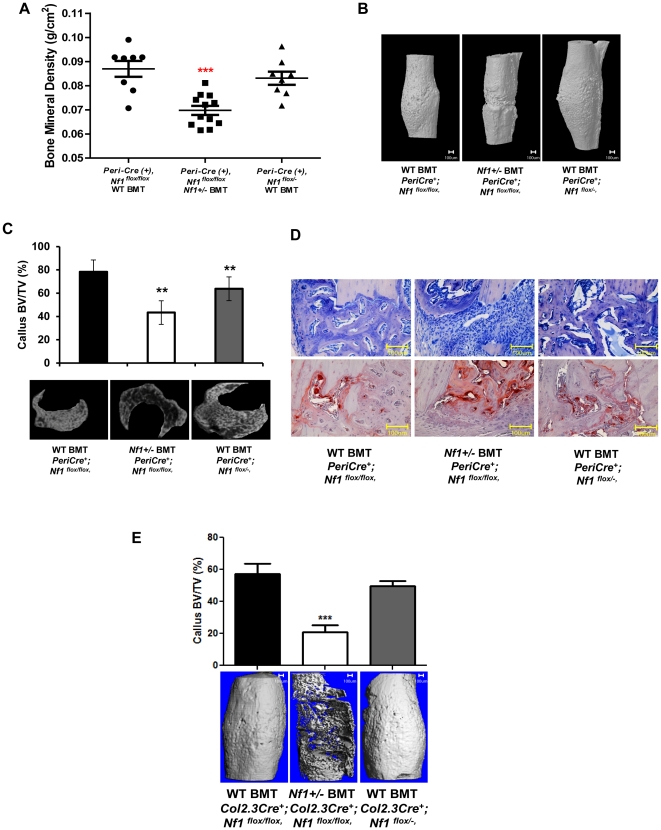
*Nf1*+/− bone marrow is necessary for skeletal deficits in *PeriCre; Nf1^flox/flox^* and *Col2.3Cre; Nf1^flox/flox^* mice. (**A**) Lethally irradiated *PeriCre; Nf1^flox/flox^* and *PeriCre; Nf1^flox/−^* mice were transplanted with *Nf1+/−* or WT bone marrow and monitored until 4 months following the transplantation. Tibia bone mineral density was measured 4 months after bone marrow transplantation (***p<0.0001). Mice then underwent tibial fracture four weeks before sacrifice. The genotypes are indicated. (**B**) 3-D reconstruction photographs from μCT scans of the fractured tibias. (**C**) Representative μCT scan callus sections from fracture sites of *PeriCre; Nf1^flox/flox^* and *PeriCre; Nf1^flox/−^* mice transplanted with WT or *Nf1+/−* bone marrow (lower panel). The upper panel provides quantitative data of the callus BV/TV ratio (**p<0.01). (**D**) Representative photomicrographs of TRACP staining (upper panels 200×) and McNeal's staining (lower panels 200×) from *PeriCre; Nf1^flox/flox^* and *PeriCre; Nf1^flox/−^* tibiae reconstituted with WT or *Nf1+/−* bone marrow. (**E**) Upper panel shows callus BV/TV ratio from *Col2.3Cre^+^* mice transplanted with WT or *Nf1+/−* bone marrow (***P<0.0001) and lower panel are 3-D reconstruction photographs of μCT scans of the fractured tibias (*Col2.3Cre^+^* mice transplanted with WT or *Nf1+/−* bone marrow).

To determine whether the transplantation of *Nf1+/−* bone marrow cells to *PeriCre^+^;Nf1^flox/flox^* mice alters fracture healing, all the recipient mice underwent 3-point bending fracture and the fracture healing process was investigated. Reconstitution with *Nf1+/−* BMMNCs resulted in a significant reduction in BV/TV as assessed by μCT (Figure 6 B–C). In addition, the fracture healing in *PeriCre;Nf1^flox/flox^* mice transplanted with *Nf1+/−* marrow was significantly impaired relative to those receiving WT bone marrow cells. This impaired fracture healing was confirmed by histological analysis (Figure 6D), which revealed that transplantation of *Nf1+/−*, but not WT BMMNCs, led to impaired fracture healing (Figure D). Similar results were obtained in a parallel study using *Nf1+/−* BMMNCs transplanted into *Nf1^flox/lfox^;Col2.3Cre+* recipients (Figure 6E). Most importantly, transplantation of WT BMMNCs restored the fracture healing of *Nf1^flox/l−^;PeriCre+* recipients (Figure 6 A–D) and *Nf1^flox/−^;Col2.3Cre+* recipients (Figure 6E). Collectively, these data indicate that the haploinsufficient bone marrow microenvironment plays an essential role in the skeletal manifestations of NF1, particularly in fracture non-union.

## Discussion

Although skeletal manifestations collectively affect up to 50% of NF1 patients [44], [45], [46], [47], the cellular mechanism(s) underlying the development of skeletal defects in NF1 patients are incompletely characterized. Previous attempts using other murine models have identified single lineage and phenotypic alterations that do not fully recapitulate the pathological phenotypes observed in individuals with NF1 [32], [33], [48], [49]. The genetic intercrosses conducted in the present study provide novel murine models (*PeriCre^+^;Nf1^flox/−^* mice and *Col2.3Cre^+^;Nf1^flox/−^* mice ) that closely recapitulate multiple skeletal phenotypes observed in NF1 patients, particularly the pathological non-union of fractures affecting young children.

In the present study, we generated two novel murine models using Cre-mediated recombination under the direction of either the MSC/osteoblast-selective periostin fragment promoter or the osteoblast-specific Col2.3 Cre promoter. In these mice, one *Nf1* allele was systemically deleted from all tissues (global knockout), while the remaining allele was conditionally deleted in mesenchymal stem/progenitor cells and/or osteoblasts. The two models allow us to compare the impact of the deficiency of *Nf1* at different stages during MSC and osteoblast development of the skeletal deficits. *PeriCre^+^;Nf1^flox/−^* mice and *Col2.3Cre^+^;Nf1^flox/−^* mice show increased osteoclast development compared to control mice *in vivo*. In addition, these mice display reduced bone mineral density (BMD) and impaired fracture healing compared to control mice.

Recent studies have begun to elucidate the interactions between haploinsufficient and nullizygous *Nf1* cells within the slow growing tumors found in individuals with NF1. Consistent with *Nf1* being a tumor suppressor gene, Zhu, et al have shown that nullizygosity of *Nf1* in the tumorigenic Schwann cell is required for plexiform neurofibroma progression [15]. However, in this model which results in a small fraction of *Nf1−/−* Schwann cells that likely appear analogous to the human situation, haploinsufficiency in non-tumorigenic lineages is also required [15]. Furthermore, Daginakatte, et al. have shown a similar phenomenon in optic gliomas in the NF1 murine model [13], [14]. Recently, we reported that haploinsufficiency of *Nf1* in the hematopoietic microenvironment specifically has a critical role in plexiform neurofibroma formation [12]. Thus, while there is a structural framework that explains the development of slow growing tumors in NF1 patients, no such pathogenic framework to explain the genesis of non-malignant conditions exists. In the present study, we showed that the full development of skeletal manifestations in the NF1 murine model requires both the loss of *Nf1* in osteoblast precursors/osteoblasts and the haploinsufficiency of *Nf1* in the hematopoietic microenvironment. This data is consistent with a single case report by Stevenson and colleagues who found that localized nullizygous loss of NF1 in pseudarthrosis tissue is acquired by micro-dissection and NF1 haploinsufficiency in peripheral blood [29].

Given that our adoptive transfer study indicates that the *Nf1* haploinsufficient bone marrow cells are important for non-union to occur and WT bone marrow cells restores fracture healing, these observations lead to opportunities of clinical and therapeutic significance. Here, we show that *PeriCre^+^;Nf1^flox/−^* mice transplanted with *Nf1+/−*, but not WT bone marrow, exhibit impaired fracture healing. Most importantly, WT transplantation of bone marrow restored fracture healing of *PeriCre^+^;Nf1^flox/−^* and *Col2.3Cre^+^;Nf1^flox/−^* mice. We previously reported a key role for *Nf1* haploinsufficiency in the elevated bone resorptive activity in both murine and human NF1 osteoclasts [31]. This lineage is likely the key effector of the microenvironment. To our knowledge, this study provides the first evidence of a non-cell autonomous mechanism for the pathologic development of non-malignant NF1 pathologic manifestations. As such, these models provide an excellent platform to further study the cellular and molecular mechanism(s) underlying skeletal manifestations in NF1 patients. Future research investigating the specific lineage and molecular targets in the microenvironment that are critical for the non-union fracture healing in NF1 is necessary.

Collectively, these data demonstrate a critical role of the hematopoietic microenvironment in fracture healing in the NF1 population. Our study provides strong evidence of a non-cell autonomous mechanism for the skeletal manifestations of NF1 and demonstrates that the hematopoietic microenvironment is instrumental for bone loss and impaired fracture healing process.

## Methods

### Ethics statement

Animal care and experiments were conducted according to the guidelines established by the Indiana University Animal Care and Use Committee (IACUC). This study was approved by IACUC via Protocol Number 0000003401 originally approved on 6/19/09.

### Animals

*Nf1+/−* mice were obtained from Dr. Tyler Jacks at the Massachusetts Institute of Technology (Cambridge, MA) [50]. *Nf1^flox/flox^* mice were provided by Dr. Luis Parada at the University of Texas Southwestern Medical Center [15]. *3.9kb^peri-EGFP/Cre^* (*PeriCre*) transgenic mice were generated as described elsewhere [38], [51]. To examine the lineage distribution of Cre-Mediated recombination, adult *PeriCre* transgenic mice were crossed with ROSA26 reporter mice. *Col2.3Cre* transgenic mice were generated as described elsewhere [52]. All studies were approved by the Institutional Animal Care and Use Committee.

### Histochemical detection of β-gal activity

To examine the Cre recombinase expression pattern in MSC and skeletal tissues, LacZ expression was determined as described previously [52]. Histochemical detection of β-gal activity was performed on adult MSCs; cultured osteoblasts; calvaria, femur, and tibiae of *PeriCre^+^;Nf1^flox/−^;Rosa^flox/flox^* mice and *PeriCre^−^;Nf1^flox/−^;Rosa^flox/flox^* mice. Tissues/cells were fixed for 3 hours in a 0.1 M sodium phosphate buffer (PBS, pH 7.3) containing 0.2% gluteraldehyde, 5 mM EGTA, and 2 mM magnesium chloride. Following 3 wash cycles with a buffer containing 0.1 M PBS, 2 mM magnesium chloride, 0.01% deoxycholate, and 0.02% NonidetP40, tissues/cells were incubated for 4 hours at 37°C with β -gal staining solution containing 50 mg β -gal (Sigma Chemical Company, St. Louis, MO, USA), 0.106 g potassium ferrocyanide, and 0.082 g potassium ferricyanide in 50 ml wash buffer. After the staining, tissues/cells were washed 3 times with PBS and placed overnight in 10% formalin. The samples were then decalcified with 14% EDTA and embedded in paraffin. 6 µm thick sections were stained for LacZ with a hematoxylin counterstain.

### Longitudinal *in vivo* peripheral dual-energy X-ray absorptiometry (pDEXA)

To evaluate the bone mass in each genotype of mice, bone mineral density (BMD) of the tibial diaphysis was evaluated *in vivo* using pDEXA (PIXImus II; GE-Lunar Corp., Madison, WI) [48]. Adult mice were anesthetized via inhalation of 2.5% isoflurane (IsoFlo; Abbott Laboratories, North Chicago, IL) mixed with O_2_ (1.5 liter/min). The mice were placed in a prone position on the specimen tray and scanned. The head was excluded from total body scans. The region of interest included the central 50% of the whole tibia.

### Micro (μ) computed tomography

Bone volume and microarchitecture in the distal femoral metaphysis were evaluated using a high-resolution desktop microcomputed tomography imaging system (μCT-20; Scanco Medical AG, Basserdorf, Switzerland). For evaluation of the trabecular envelope at the distal femoral metaphysis, each specimen was scanned with a slice increment of 9 µm. CT images were reconstructed, filtered (σ = 0.8 and support = 1.0), and thresholded (22% of maximum possible gray scale value) as previously described [53]. Scanning for the femur was started at 15% of the total femur length measured from the tip of the femoral condyle and extended proximally for 200 slices. The area for trabecular analysis was outlined within the trabecular compartment, excluding the cortical and subcortical bone. Every 10 sections were outlined manually, and the intermediate sections were interpolated with the contouring algorithm to create a volume of interest. Parameters of microarchitecture for both skeletal sites included bone volume (BV, mm^3^) and bone volume fraction (BV/TV, %), as well as trabecular number (Tb.N, mm^−1^), trabecular thickness (Tb.Th, µm), and trabecular separation (Tb.Sp, µm).

### Fluorochrome bone labeling and histomorphometric measurements

Fluorochrome labeling of the bones was performed in 8 week-old mice by intraperitoneal injections of calcein (20 mg/kg, Sigma Chemical, St. Louis, MO, USA) and alizarin (20 mg/kg, Sigma) 8 and 3 days before sacrifice, as previously described [54]. After sacrifice, femurs were fixed in 10% neutral buffered formalin for 48 hours, dehydrated in graded ethanols, and embedded undecalcified in methylmethacrylate. Frontal sections (4 µm thick) were cut from the distal femur using a motorized microtome equipped with a tungsten-carbide knife (Leica Inc, Deerfield Il). The sections were reacted with tartrate-resistant acid phosphatase (TRACP) for identifying and measuring the osteoclast surface or mounted unstained for fluorochrome-derived bone formation parameters. One section per tibia was viewed at 160× magnification on a Leitz DMRXE microscope (Leica Mikroskopie und System GmbH, Wetzlar, Germany) and the image captured using a SPOT digital camera (Diagnostic Instruments, Inc., Sterling Heights, MI). The measurement area for the distal femoral metaphysis was determined by a region beginning 0.5 mm proximal to the midpoint of the growth plate, non-inclusive of cortical bone, and extending proximally for a total area of approximately 2.8 mm^2^. Trabecular bone turnover was assessed by measuring the extent of single label (sL.Pm), double label (dL.Pm) and the area of bone (dL.Ar) between the calcein and alizarin labels using Image Pro Plus version 4.1 software (Media Cybernetics, Silver Spring, MD). Derived histomorphometric parameters included mineralizing surface (MS/BS, %), a measure of active bone-forming surface, calculated as follows: the [½ sL.Pm+dL.Pm]/Tt.Pm * 100; mineral apposition rate (MAR, µm/day), a measure of the rate of radial expansion of new bone, calculated as follows: dL.Ar/dL.Pm/4 dy; and bone formation rate, an overall measure of bone formation that combines MS/BS and MAR, calculated as follows: MS/BS * MAR * 3.65.

For histological analysis of cell populations, after sacrifice, tibiae were dissected from the mice and were fixed in 10% neutral buffered formalin for 48 hours, dehydrated in graded ethanols, and embedded undecalcified in methylmethacrylate. Frontal sections (4 µm thick) were cut from the tibiae using a motorized microtome equipped with a tungsten-carbide knife (Leica Inc, Deerfield Il). The sections were reacted with TRACP for identification and measuring of osteoclast surface. MacNeal and /or von Kossa staining were utilized for identifying osteoblasts and calcified bones. The sections were viewed at 200× magnification on a Leitz DMRXE microscope (Leica Mikroskopie und System GmbH, Wetzlar, Germany) and the images were captured using a SPOT digital camera (Diagnostic Instruments, Inc., Sterling Heights, MI). The quantification of osteoblast cell number and osteoclast surface were normalized to the bone surface in millimeters (mm), to control for the total amount of trabecular bone surface within each histological section.

### Generation of fracture and X-ray scan

A single tibial fracture was induced as described previously [55], [56], [57]. With the animal lying supine on the operating table, an intramedullary rod (a 30-G needle) was surgically inserted via the proximal end of the tibiae into the medullary canal of the tibia, so as to stabilize the bone post-fracture and permit load-bearing. A 200-gram weight was dropped from a height of 30-cm to fracture the tibia between the middle to lower 1/3 of the bone. Mice were X-rayed following the fracture to verify standardization of the fractures. The mice were allowed to recover on a heated pad and then placed in recovery cages. Fracture repair was monitored by weekly radiography (pixRay-100, Bioptics, Phoenix, AZ) and cases where the internal fixation failed, (due to pin slippage, bending or breakage) the affected mouse was euthanized and excluded from subsequent analysis.

### MSC and committed osteoprogenitor culture in murine specimens

MSCs were generated from each experimental group of mice as previously described [49]. Briefly, BM mononuclear cells (BMMNCs) were separated by low density gradient centrifugation from 6∼8-week old mice. BMMNCs were then resuspended and cultured in mouse MesenCult basal medium containing MesenCult Supplement (Stem Cell Technologies Inc.) at a concentration of 2×10^6^ cells/mL in a 10-cm tissue culture plate as previously described. When the cultures reached 80–90% confluency, cells were trypsinized and replated at a concentration of 5×10^5^ cells/75 cm^2^. MSCs at passage 5 to 10 were used for the following experiments. To culture committed osteoprogenitors, calvaria or femur distal metaphyses were resected and chopped finely with a scalpel. Bone chips were exposed to 0.25% trypsin for 1 hour and then passed through a 70-µm cell strainer (Falcon, Franklin Lakes, NJ, USA) to remove debris and matrix remnants. Cells were cultured in α-MEM medium and supplemented with 10% fetal bovine serum (FBS) and 1% penicillin–streptomycin at 37°C, 5% CO_2_.

### Bone marrow transplantation of hematopoietic cells

10^6^ syngeneic WT or *Nf1+/−* bone marrow cells from WT GFP or *Nf1+/−* GFP mice were transplanted into young adult *PeriCre*;*Nf1^flox/flox^* recipient mice following 1100 rads of ionizing radiation with two split doses [12]. The C57BL/6-TgN(ACTbEGFP) 1Osb transgenic mouse line was obtained from Jackson Laboratory, Bar Harbor, Maine [58].

Statistical analysis. One way ANOVA and post *t*-test was used for all the statistical analysis in this study. P values less than 0.05 are considered as statistically significant.

## Supporting Information

Figure S1 ***PeriCre*** **is specifically expressed in the skeletal tissues of adult** ***PeriCre*** **transgenic mice.** (**A**) Four-week old *PeriCre* transgenic (b,d) or control (a, c) mice were dissected and fixed in 4% paraformaldehyde. The whole bodies were washed in β-gal buffer 3×15 minutes at 4°C and then overnight with shaking. After a final wash in β-gal buffer, the whole bodies were stained with β-gal substrate at 37°C for 3 hrs. Periostin-Cre expression is reflected by blue LacZ staining. Representative cross sections from LacZ staining with H&E counterstaining are shown. *PeriCre* expression is reflected by blue staining. (**B**) Osteoblast cell cultures were prepared by digestion of the calvaria and incubated in alpha-MEM medium supplemented with 10%FBS, ascorbic acid, β-galactophospate, and dexathamosone. MSCs were prepared from bone marrow mononuclear cells. The cells were fixed in 4% paraformaldehyde in PBS and washed in β-gal buffer 3×15 minutes at 4°C. Cells were stained in β-gal substrate at 37°C for 3 hours. Blue staining represents LacZ positive osteoblasts or MSCs. (**C**) Neurofibromin expression in WT, *Nf1+/−*, and *Nf1−/−* MSCs and calvarial osteoblasts evaluated by western blot.
(TIF)

Figure S2 **PCR of tail DNA.** Differential expression of *Nf1*, *PeriCre*, *Loxp*, and recombinant *Cre* was confirmed by PCR.
(TIF)

Figure S3 ***PeriCre^+^;Nf1^flox/−^*** **mice exhibit reduced body size and weight.** (**A**) Photographs of representative 4 month old mice from each genotype. (**B**) Body length was measured from the snout to the proximal end of the tail in 4 month old mice of each genotype. The number of animals analyzed were WT, n = 24; *Nf1+/−*, n = 20; *PeriCre^+^;Nf1^flox/flox^*, n = 27; *PeriCre^+^;Nf1^flox/−^*, n = 48. (**C**) Quantitative data of body weight of 4-month old mice of each genotypes.
(TIF)

Figure S4 The donor cells are presented in recipient mice. (**A**) Flow cytometric analysis of peripheral blood shows more than 95% cells are GFP positive. Colonies were observed under light microscope and fluorescent microscope. 100% of colonies are GFP positive. (**B**) Individual colonies were picked up from methylcellulose cultures and were subjected to PCR analysis.
(TIF)
