# Supplementary figures and images for: The Haploinsufficient Hematopoietic Microenvironment Is Critical to the Pathological Fracture Repair in Murine Models of Neurofibromatosis Type 1
Source: PLoS One. 2011 Sep 29;6(9):e24917. doi: 10.1371/journal.pone.0024917 (PMC3182976; doi:10.1371/journal.pone.0024917)

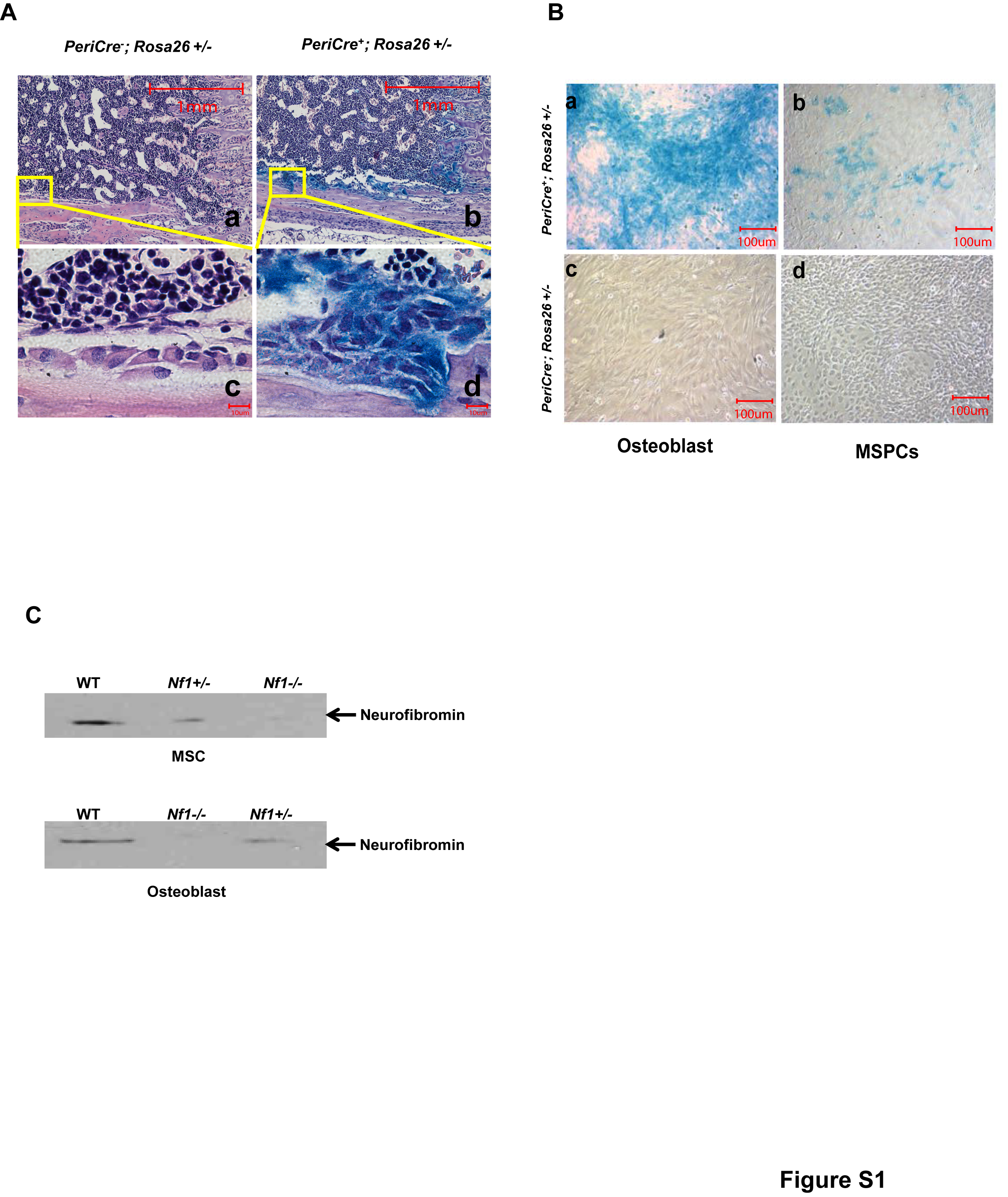

Supplement: Figure S1 — PeriCre is specifically expressed in the skeletal tissues of adult PeriCre transgenic mice. (A) Four-week old PeriCre transgenic (b,d) or control (a, c) mice were dissected and fixed in 4% paraformaldehyde. The whole bodies were washed in β-gal buffer 3×15 minutes at 4°C and then overnight with shaking. After a final wash in β-gal buffer, the whole bodies were stained with β-gal substrate at 37°C for 3 hrs. Periostin-Cre expression is reflected by blue LacZ staining. Representative cross sections from LacZ staining with H&E counterstaining are shown. PeriCre expression is reflected by blue staining. (B) Osteoblast cell cultures were prepared by digestion of the calvaria and incubated in alpha-MEM medium supplemented with 10%FBS, ascorbic acid, β-galactophospate, and dexathamosone. MSCs were prepared from bone marrow mononuclear cells. The cells were fixed in 4% paraformaldehyde in PBS and washed in β-gal buffer 3×15 minutes at 4°C. Cells were stained in β-gal substrate at 37°C for 3 hours. Blue staining represents LacZ positive osteoblasts or MSCs. (C) Neurofibromin expression in WT, Nf1+/−, and Nf1−/− MSCs and calvarial osteoblasts evaluated by western blot. (TIF) [file pone.0024917.s001.tif]

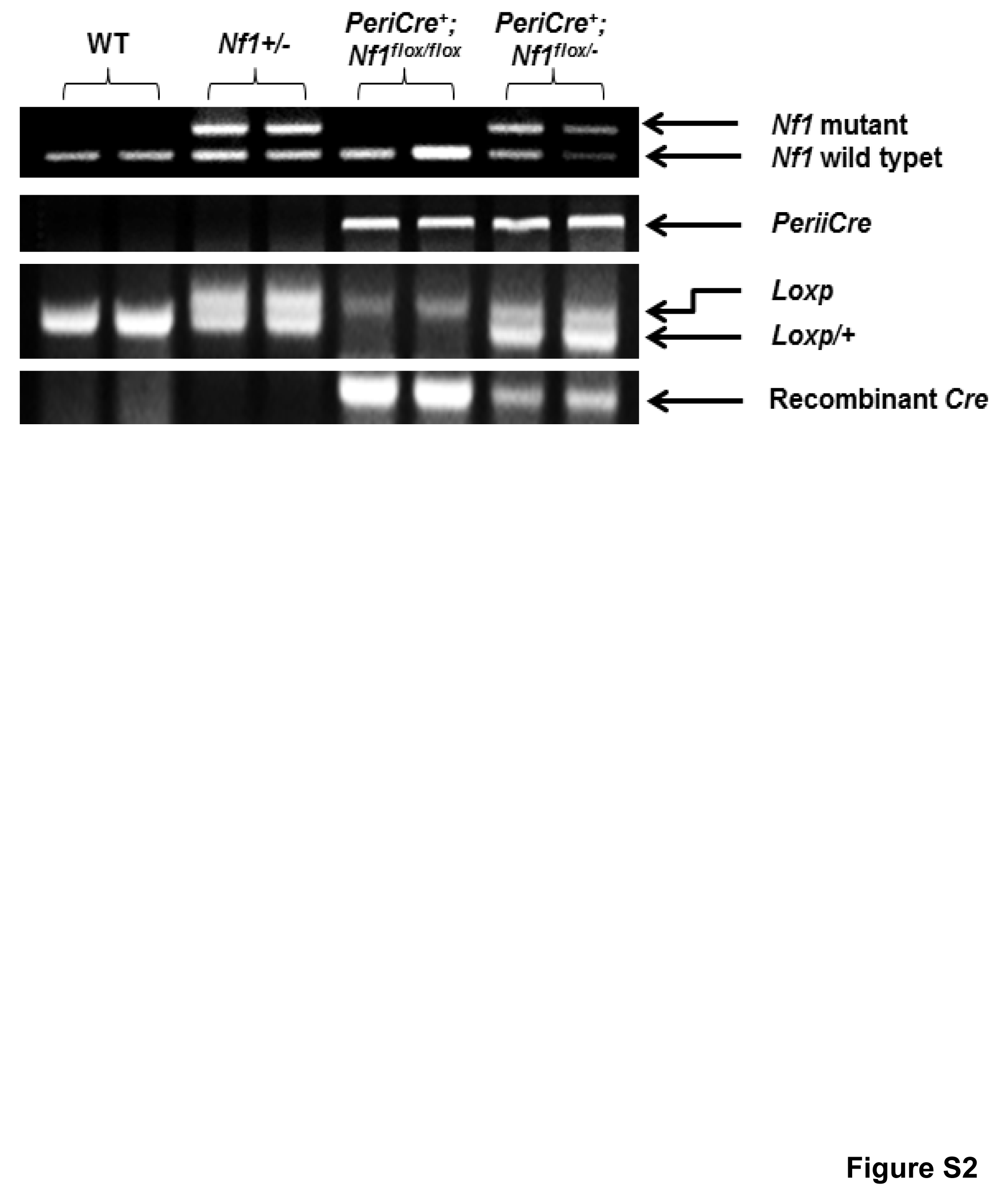

Supplement: Figure S2 — PCR of tail DNA. Differential expression of Nf1, PeriCre, Loxp, and recombinant Cre was confirmed by PCR. (TIF) [file pone.0024917.s002.tif]

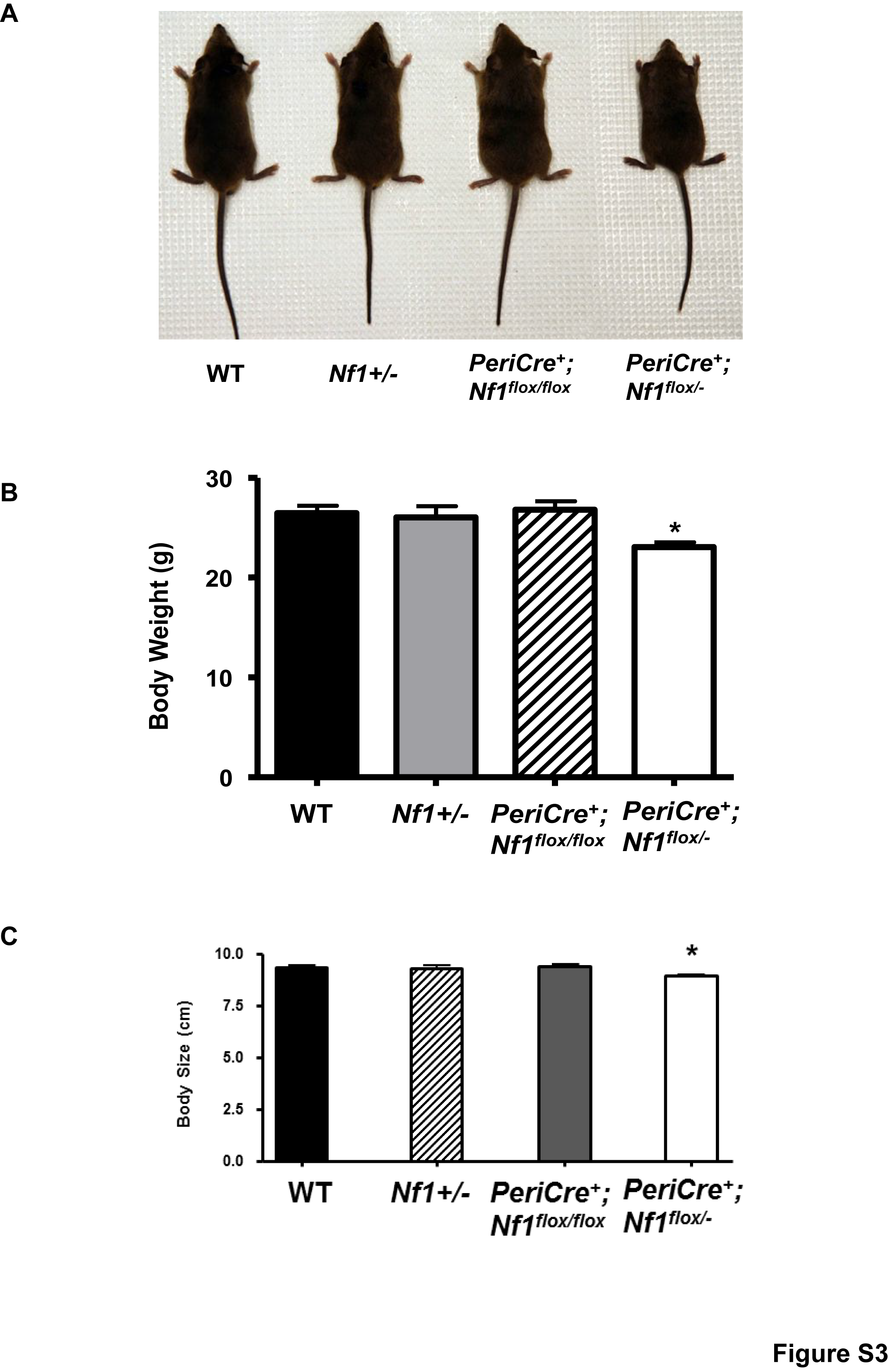

Supplement: Figure S3 — PeriCre+;Nf1flox/− mice exhibit reduced body size and weight. (A) Photographs of representative 4 month old mice from each genotype. (B) Body length was measured from the snout to the proximal end of the tail in 4 month old mice of each genotype. The number of animals analyzed were WT, n = 24; Nf1+/−, n = 20; PeriCre+;Nf1flox/flox, n = 27; PeriCre+;Nf1flox/−, n = 48. (C) Quantitative data of body weight of 4-month old mice of each genotypes. (TIF) [file pone.0024917.s003.tif]

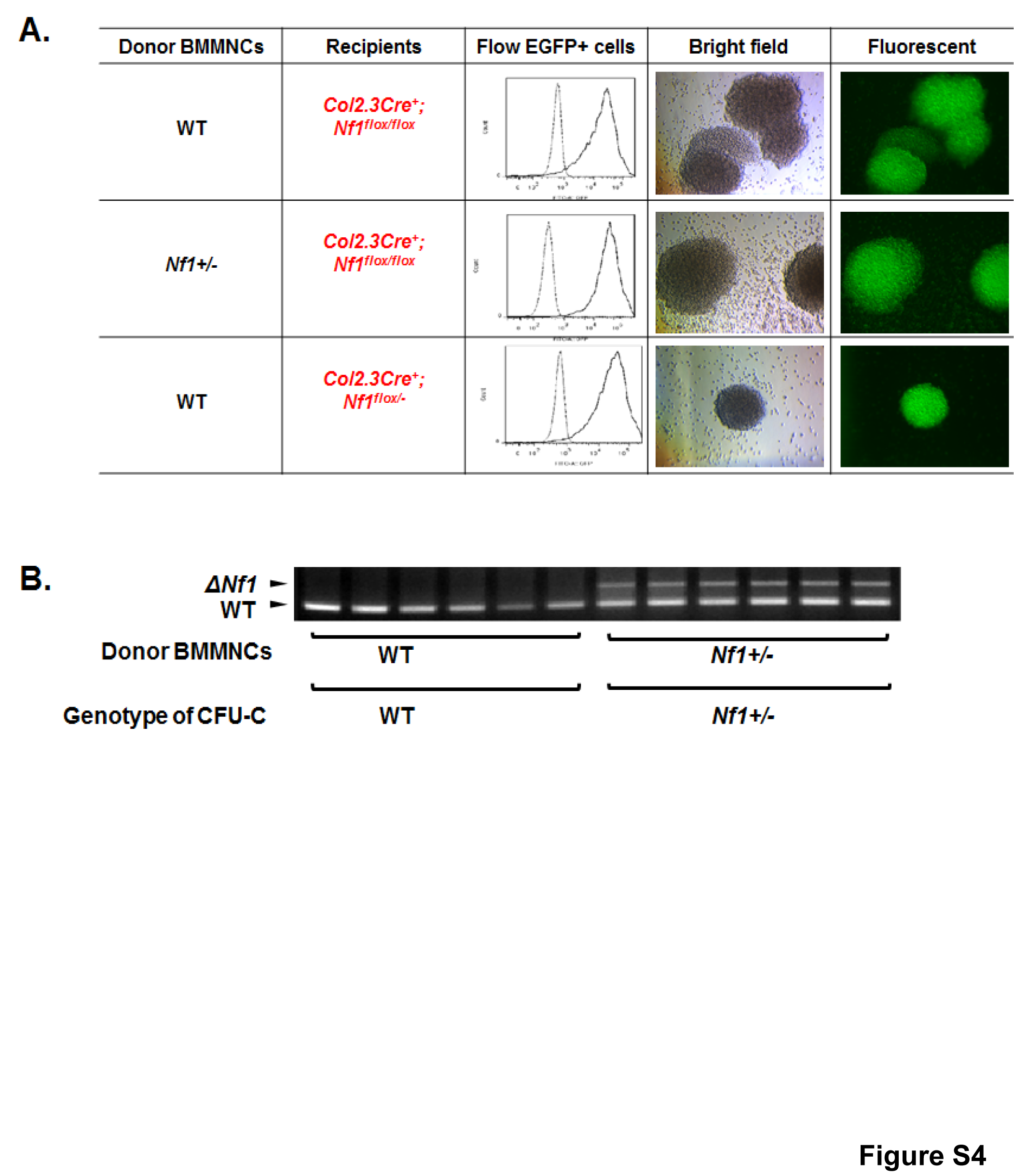

Supplement: Figure S4 — The donor cells are presented in recipient mice. (A) Flow cytometric analysis of peripheral blood shows more than 95% cells are GFP positive. Colonies were observed under light microscope and fluorescent microscope. 100% of colonies are GFP positive. (B) Individual colonies were picked up from methylcellulose cultures and were subjected to PCR analysis. (TIF) [file pone.0024917.s004.tif]
